# Sustainability as an Intrinsic Moral Concern for Solidaristic Health Care

**DOI:** 10.1007/s10728-023-00469-5

**Published:** 2023-09-04

**Authors:** Marcel Verweij, Hans Ossebaard

**Affiliations:** 1 Ethics Institute, Utrecht University, Janskerkhof 13, 3512BL, Utrecht, The Netherlands; 2 Free University, Amsterdam, The Netherlands; 3 National Health Care Institute, Diemen, The Netherlands

## Abstract

Environmental pollution and greenhouse gas emissions that contribute to climate change have adverse impacts on global health. Somewhat paradoxically, health care systems that aim to prevent and cure disease are themselves major emitters and polluters. In this paper we develop a justification for the claim that solidaristic health care systems should include sustainability as one of the criteria for determining which health interventions are made available or reimbursed – and which not. There is however a complication: most adverse health effects due to climate change do occur elsewhere in the world. If solidarity would commit us to take care of everyone’s health, worldwide, it might imply that solidaristic health system cannot justifiably restrict universal access to their own national populations. In response we explain health solidarity is to be considered as a moral ideal. Such an ideal does not specify what societies owe to whom, but it does have moral implications. We argue that ignoring sustainability in political decision making about what health care is to be offered, would amount to betrayal of the ideal of solidarity.

## The Environmental Impact of Health care

Ecological crises rage the world and adverse consequences on health and health care become more and more obvious. Vital planetary boundaries are already being transgressed and scientists fear this will have irreversible impacts on the ecological systems that support life on earth [1, 2]. Many scientists who contributed to the latest report of the Intergovernmental Panel on Climate Change (IPCC) expect the world to warm by at least 3 °C by the end of the century, much more than the target of 1.5 to 2 degrees Celsius as set in the UN Paris agreement [3]. The unrestrained use of fossil fuels leads to rising temperatures, more extreme weather events and rising sea level, that affect the lives of millions of people worldwide. The evidence is mounting and consequences of climate change can be witnessed by all inhabitants of the planet, scientists and lay-people alike. Loss of biodiversity, the pollution of water, air and soil, the changing course of biogeochemical cycles represent further degradations of the biosphere with equally destabilizing effects [1].

Most countries observe increasing climate-related health care demands particularly concerning cardiovascular disease, infectious diseases (zoonoses), mental health and respiratory diseases [4]. In low resources countries with fragile health care systems outcomes are especially poor for the most vulnerable: babies and children, mothers, minorities, the poor, the elderly. Forced migration, displacement, conflict, undernutrition, food and water insecurity are additional context-specific consequences that affect these groups. The alarm bell is sounding and countries now close green deals and climate agreements to collaborate to face these enormous challenges. All sectors in society must reduce their greenhouse gas emissions in faster ways in order to achieve the climate objectives that their governments committed to. Mitigation and adaptation measures are taken to make consumption and production processes more sustainable, i.e. to generate a smaller ecological footprint in terms of environmental and climate impact [5].

This also applies to the health care sector itself, which accounts for at least 5% of total CO_2_ emissions worldwide [6]. At the UN climate change conference in Glasgow, November 2021, fifty countries committed to actively decarbonize their health care systems. Healthcare professionals have also become more and more aware of the urgency and relevance to reduce waste and pollution and invent ways to reduce both consumption of care (e.g., by means of primary prevention) and production of care (e.g., by avoiding overtreatment). Such awareness is not uncommon to the health care sector [7] although the role of health care as a polluter and carbon emitter has reached the environmental ethics and bioethics community only recently [8, 9].

There is an obvious necessity, and arguably a strong moral and legal^1^ obligation for societal institutions (governments, municipalities, schools, companies, etc.) - and for all citizens to adopt more sustainable practices and to reduce pollution and greenhouse gas emissions. At first sight, one might think that health care is not different in this compared to any other sector. Moreover, one might even assume that the burden of responsibility to reduce emissions is better allocated to *other* sectors: public health care is an essential good in any flourishing society, hence it might be reasonable to tolerate some negative environmental impact from health care interventions, while imposing much stronger sustainability requirements on, for example, the automotive industry, air traffic, or the production and use of luxury products.

Although we do think it makes perfect sense to put much more pressure on such other sectors to reduce greenhouse gas emissions and other forms of pollution, in this paper we argue that for health care systems sustainability is an *intrinsic* moral concern in a way that it for not in most other sectors. Our analysis specifically concerns health care systems with universal coverage, which presuppose and enact solidarity between citizens. If environmental sustainability is an intrinsically relevant concern in the practice of medicine and health care, this means that it should play a role in judgements about what good care involves and what medical treatments should be offered, together with other intrinsically relevant considerations, like medical effectiveness, safety, and patient-centeredness of interventions.^2^ Our central claim is that solidaristic health care systems should endorse environmental sustainability as an intrinsic consideration in regulating health care provision and resource allocations: sustainability must be taken into account in decisions about which health care provisions are included in universal health insurance or public health care. This can imply that for specific conditions a more sustainable but somewhat less effective medical treatment is to be preferred to a more effective but also more polluting alternative. Trade-offs will be inevitable.

How to understand and assess ‘sustainability’ is itself a matter of controversy, for example to the extent in which it includes social and economic aspects, or in the role that is given to considerations of intergenerational or global justice [10]. Moreover, there are many different ways in which activities can be considered as environmentally unsustainable. In this paper we will sidestep conceptual discussions and focus specifically on sustainability in the sense of climate neutral activities, hence specifically explore arguments for health care to reduce greenhouse gas emissions. Obviously, other forms of environmental pollution such as antibiotics spoilage also have negative health impacts – often even much clearer and direct as they occur in the local environment. Yet the case for sustainability as intrinsic concern for health care raises specific complexities if it is about CO_2_ emissions and climate change, as these primarily affect populations in other parts of the world. If we succeed in answering these complexities, this offers strong support for the broader claim that health care provision should aim to reduce or avoid *any* environmental damage that negatively impacts health.

## Sustainability as an Intrinsic Moral Concern in Health care Systems?

It may seem as if there is a straightforward case for considering sustainability an intrinsic concern for health care institutions. Environmental damage and climate change are major determinants of disease everywhere in the world and therefore health care systems that aim to protect health and seek optimal health outcomes, should try to reduce causing environmental damage and greenhouse gas emissions. Human health depends on the health of the planet, so protecting the planet is to protect ourselves [11]. What else is needed?

An initial complexity is that more sustainable options may also be less effective, more expensive or less safe. Examples are the use of disposables and hygienic plastic packages: these are arguably less sustainable than some alternatives, but they may prevent contaminations and increase safety. Choosing for more sustainable options could thus lead to poorer health outcomes. Now at first sight this is easily resolvable: all medical interventions have a risk of adverse effects for patients and therefore those risks need to be balanced against the expected benefits of the treatment. By determining the overall health impact of different treatment options (effects of treatment minus negative health outcomes due to environmental impact of treatment) we can choose for the option that maximizes health. Munthe et al. argue that in allocation decisions, negative dynamic effects on health should be taken into account, and this supports more sustainable choices [12]. To this extent Polisena et al. refer to the importance of introducing environmental aspects in Health Technology Assessment procedures [13].

However, this leads to a second problem, namely that environmental health impacts will often not specifically occur in the target population or society of a particular health system. Climate change is a worldwide problem, and the negative impact of unsustainable activities here and now will not be local but contribute to the global problem. Moreover, the largest burdens of climate change will be borne by populations in the global south, and by future generations. On the other hand, the primary objective of health care systems is to promote and protect health of *their own* population. This also applies to health systems that are explicitly based upon notions of solidarity, like in, for example, the Netherlands, United Kingdom, or Canada. This can be seen as a weakness of notions of solidarity – they seem to presuppose a particular group to which solidarity is constrained. Indeed, notions of solidarity as endorsed in health policies and health care systems are often relatively thin: “we” as a (national) society share costs and risks of health and disease, which specifically implies solidarity between rich and poor, between the young and the old, and between the healthy and the sick – but only within “our” society.

This limitation of solidaristic health systems again suggests a straightforward response, namely that, from an ethical and thus impartial perspective, solidarity should *not* be constrained to one’s own group; instead, solidarity should aspire to be universal and global. There is much to be said for that, given the immense inequalities in access to high-quality medical care between high-income and low-income countries.

Now, as attractive as this looks, it triggers a further complication. After all, *if* health solidarity is to be understood as going beyond national boundaries, and *if* the same notion of solidarity is the basis of many national health care systems, this would imply that health care systems should also serve and seek optimum *global* health. As a consequence, health systems in high income countries should offer access for any patient in need, irrespective of where they live. If this is practically and politically feasible at all, the health care that could be offered in such an entirely global and universal system would be much more sober than it is now in high-income countries.^3^ One might consider this an attractive ideal from a global health perspective, but it does not help to solve the problem we are facing now, namely: should high income health care systems see sustainability as an intrinsic concern, and if so, how should that affect their resource allocations?

## How Solidarity can Ground Obligations

Can we make sense of solidarity in such a way that it supports embracing the global health impact of greenhouse gas emissions as an intrinsic concern for health care systems, without also implying that those systems should offer access to anyone – irrespective of where they live?

Since the Nuffield Council on Bioethics published Prainsack and Buyx’ study, [14] the concept of solidarity has become much more central to ethical and philosophical discussions in health care. These discussions at least show the complexity and illusiveness of the concept. Solidarity is not a general moral principle like ‘respect for autonomy’ or ‘non-maleficence’, in which the core meaning can be considered as a relatively clear general norm for citizens, professionals or organizations [15]. Solidarity is much more a description of desirable practices and acknowledgement of a sense of belonging together, than a clear statement of obligation that applies to everyone. The combination of descriptive and evaluative dimensions adds to the complexity, if only because this suggests that ‘solidarity’ only has normative implications for people who already see themselves as member of a group with mutual bonds. This raises questions as to how solidarity can result in obligations if this shared sense of belonging itself is weak or absent, and questions about who belongs to the group in the first place [16].

The most general or basic idea of solidarity is that of people standing together and assist each other in the face of threats, acknowledging a connection or some similarity between them [17]. The sense of connection will often be linked to the nature of the threats themselves: they originate from a common enemy, or from the realization that we are all vulnerable to some disease, or from our understanding of how wellbeing (or health) of people is interconnected.

To confront threats together, implies, for example, jointly bearing costs of protection of those who are especially vulnerable. This attitude can be desirable for different reasons – either as a prudential choice: we have a common interest, and each of us has self-interested reasons to participate in a reciprocal practice [16]. Or because we have strong a sense of community with existing mutual commitments and care, which lead us to view threats to some individual group members as a danger to all of us [18, 19]. The latter sense of belonging could also be understood as an extension of basic benevolence from one individual to another [17].

Now, the more solidarity is shared, embedded and practiced in a society, the more it can also be institutionalized in a system (e.g. health insurance) that includes rights and obligations for citizens – and these will be governed by other notions, such as fairness, or the specific arrangements that are the outcome of political decision making [17, 20]. Such institutions may even be necessary to sustain the more spontaneous solidaristic practices. The overall moral notion of solidarity as standing together, as such does not offer any guidance about *who* belongs to the group, and *what* exactly each of us, or we jointly as society, owe to others. And it does not offer guidance for setting priorities for whom to protect and to what extent, if many are threatened. Solidarity is at best an inspiring, motivating and necessitating moral imperative that urges us to ‘stand together’, but it does not explain what exactly is required, of whom. We need to build further political arrangements, inspired by solidarity, that do offer such guidance; and such arrangements, like any other institution in a democratic context, will be constrained by notions of justice and fairness.

In a health insurance system, this may, for example, imply that it is the people who have paid premiums for health insurance who have access, and that all inhabitants of a country are obliged to buy insurance, like in the Netherlands [20]. Something similar will apply to national health care systems based on taxes, such as the UK NHS. The obligation to pay one’s fair share of premiums or taxes in a national system can only be imposed on the inhabitants, and this restricts the circle of solidarity. In this way the solidaristic health system is also reciprocal, and most citizens can rightly consider participating in this joint endeavor to be a prudential ‘choice’. There might be no real (freedom of) choice because, to ensure a sharing of costs and risks between rich and poor, between low-risk and high-risk populations, and between the healthy and sick, a solidaristic system may need to be *imposed on all* – and not only on those who are in need. Hence, only when solidarity is institutionalized it becomes possible to specify what we owe to each other.

This also resolves the last problem as presented in the previous section. *If* solidarity in health care is seen as a moral reason to care for sustainability in ‘our’ health care system, this as such does not imply the conclusion that solidarity then also requires offering everyone access to the health care provided. Who has, and who does not have access to care, and to what extent, is not determined by the notion of solidarity itself. The same applies to decisions about the contents of the health care package: what health care provisions are to be offered in national health services or reimbursed in a health insurance system? Those issues are determined by political arrangements governed by notions of efficiency, equity and procedural fairness.

So, now we have taken away a possible obstacle for linking solidarity and sustainability, what room is left to develop a positive argument for that link? How to develop the argument *for* sustainability? For this we need to understand how solidarity does function as a normative, action guiding concept after all.

## Solidarity as an Ideal

We have already suggested to see the basic notion of solidarity not as a moral principle that guides or undercuts specific obligations and moral claims in health care. But if solidarity is not itself a moral principle that can offer specific guidance as to what is owed to whom, then how to understand the normative dimensions of the concept? Solidarity is certainly more than just a description of a practice of people ‘standing together in the face of threats’;  at least it describes practices that are considered morally worthwhile, desirable, and worthy of protection.

A fruitful way to grasp solidarity, both its descriptive and normative meaning, but also the apparent lack of concrete obligatory implications, is to see it as a *moral ideal*. Ideal concepts like “democracy”, “peace”, or “solidarity” portray desirable states of affairs and ways of living worth pursuing. They are essentially future-oriented and thus offer a perspective on our current situation, but compared to practical goals or objectives, ideals are much more open and abstract. An important dimension of ideals is that they can’t be fully realized, [21] and this adds to their inspiring and action-guiding role: ideals beckon to look beyond current obstacles to progress. Note however that, if ideals can’t be realized completely, this is not because they are necessarily unrealistic, unfeasible or unpractical. What is driving the ‘unattainability’ of ideals is that, when we succeed in realizing some important elements of an ideal, we will discern new aspects still left to be pursued – just like a shifting horizon: progress leads to new outlooks and insights [22]. It implies that ideals are elusive concepts that cannot be grasped and circumscribed completely. We do have a relatively clear sense of what a perfect democracy entails, but the more we succeed in realizing it, the more we will also acknowledge that the initially projected view was not yet perfect: the ideal has shifted further, enabling critique on current institutions, and motivating further progress.

The same story applies to solidarity as an ideal for health care systems: it offers guidance for developing institutional arrangements that regulate access to health care, but the ideal of solidarity also provides a horizon against which we can discern the shortcomings of such arrangements. Ideals inspire and motivate but their normative guidance is not to prescribe or prohibit specific types of action. They may ground or motivate specific institutional arrangements – such as a health care system – but the specific rights and duties that govern those arrangements cannot be derived directly from the ideal as such. Yet solidarity as a moral ideal is still powerful, offering a normative perspective on those institutional arrangements, even if these are already satisfying certain standards of justice [22, 23]. This understanding of solidarity as ideal can account for both Dawson and Jennings’ idea that “solidarity is a value that supports and structures the way we in fact do and ought to see other kinds of moral considerations”, [19] but also for seeing solidarity as the “putty of justice”, as Prainsack and Buyx, and Kolers do: a concept that can help filling in the gaps left by other abstract notions like health justice, and offer a perspective on how these can lead to moral judgments about concrete situations [17, 23].

## Why Solidaristic Health care Systems cannot Neglect Sustainability

The moral ideal of solidarity that guides health care systems goes beyond the concrete solidaristic arrangements that regulate access to public health care or the distribution of health care costs in a specific society. Applied to health care, we suggest that the ideal posits that health is something that, in several ways, connects and is shared by all people. First, in the normative sense that we can mutually acknowledge that health is a fundamental and constitutive element for human flourishing – this holds for any human being [24]. A second sense in which health is shared is that, although the inequalities in risk are immense, ultimately each person’s health is inherently vulnerable to threats of disease. And a third is that most health determinants and risks are shared: we are interconnected due to the spread of infectious diseases, via the social and societal determinants of health, and through our shared ways of living. These mutual connections are important reasons for ‘standing together’ in solidarity, and to develop and maintain health systems that offer equal access to necessary health care for all citizens, and to jointly prevent disease within our societies.

Yet the same ideal of solidarity can indicate shortcomings and lacunas of such systems. One such shortcoming concerns the health of other people, elsewhere in the world, or of people in the future, who are not, or not yet part of ‘our’ health care system. The solidaristic acknowledgement that health is essentially shared supports (national) public health care systems offering equal access, but it is, simultaneously, at odds with the fact that access to health care is largely limited to current inhabitants of a country. That people elsewhere and in the future can’t have access to ‘our’ health system may be largely inevitable. But if ‘their’ health needs are also aggravated due to choices we make in ‘our’ system, and if we do not take those effects into account in the way we evaluate ‘our’ health care interventions (e.g. in cost-effectiveness assessments) this shows our system is actually *indifferent* about the health needs elsewhere. Such indifference is not merely in tension with solidarity, it is a negation if not betrayal of that ideal, especially if we consider that health in those populations is much more vulnerable than ours.^4^

In this way, the idea that health is something we share, and that we should ‘stand together’ to protect people who are most vulnerable, is a direct moral ground for health care systems to reduce and preferably avoid health-damaging pollution and greenhouse gas emissions that cause global warming. And maybe even stronger so if the harms of such emissions mostly affect populations that are themselves not part of that particular health care system: if the negative health impact of pollution would primarily affect people who have access to that health system, as might happen with spoilage of antibiotics in sewerage systems contributing to antibiotic resistance, [25] it is less obvious that their health needs are completely ignored by the health system – after all, they have access to care. Therefore, this would not be a betrayal of the ideal of solidarity.

We should prevent our health care systems here and now contributing to disease elsewhere, and in the future. This is not an external consideration that can or should be imposed on health care. Instead, the ideal of solidarity requires us to see sustainability as *intrinsic* to the core goals and values of the health care system. Taking global health into account in this way is certainly not unfeasible or undermining a high-quality national health care systems in high income countries. It does require making efforts to effectively reduce greenhouse gas emissions and other environmental pollution, not only in day-to-day activities in hospitals and other health care practices, but especially also in the more basic policies that determine what medical treatments and care activities are being offered in the health care system, and what standards of care are to be maintained. Sustainability should thus become a core concern for resource allocation and for setting standards for quality of health care, next to, for example cost-effectiveness, medical need, and patient-centeredness.

Some work has been done already to frame environmental and climate impact of health care delivery as one of the dimensions of health care quality [26]. There are many ways to make existing health care practices and interventions more sustainable, and this can be embedded in quality improvement measures, for which many concepts, processes, education and accreditations already exist [27].

What about resource allocation and policies that determine which treatments are to be made available and be reimbursed, and which not? How to include sustainability as consideration in these decision processes? This question goes beyond the scope of our paper, but we can at least sketch some options.

In case of interventions with a very large carbon footprint it makes of course sense to look for alternative, more sustainable interventions, for example by prioritizing prevention to care, or in some cases to preferring interventions with a smaller carbon footprint [28, 29].

Sustainability can also be considered as a factor in the assessment and appraisal of existing or novel treatments, to be weighed against other relevant considerations like medical need, effectiveness, and cost. Such weighing can be included in the calculation of cost-effectiveness of interventions, by including the costs of adverse environmental and climate health effects in the assessment of overall health benefits.

A third approach is to consider the environmental impact of health care interventions as an independent value (or ‘societal burden’) to be weighed in the resource allocation process against effectiveness, cost-effectiveness and medical need. It might also make sense to combine this with the previous approach, given that the societal costs of environmental damage and greenhouse gas emissions are not limited to adverse health outcomes. Obviously, much more work is needed, to develop appropriate procedures and methods assessing sustainability and including it in resource allocation decision making.

## Conclusion

Our analysis offers a basic justification for seeing sustainability, and especially the reduction of greenhouse gas emissions, as an intrinsic concern for solidaristic health systems, which should be taken into account in decisions about the content of basic health care packages and about quality of care standards. Both policy areas are centered around a commitment to promote and protect health, and if we take the ideal of health solidarity seriously, we can’t exclude negative health impacts that affect a population and an environment that is larger than ours.

The argument can be much more straightforward for health-care-caused pollution that primarily has a local health impact: obviously health economic approaches that determine cost-effectiveness of an intervention should include all health effects within the society in which the intervention is offered. A separate argument appealing to the broader ideal of solidarity is not necessary to support such inclusion.

It may be clear that, taking sustainability seriously will create new ethical questions. There will be tradeoffs between serving interests of patients here and now, and protecting the health of people elsewhere, or in the future. Eventually this will have impact on what is on offer. If sustainability is indeed an intrinsic concern for determining what good care is, we may have to accept that, sometimes, a treatment that is more sustainable but somewhat less effective or comfortable for patients, is to be preferred to treatments that are deemed best for the patients themselves.

## Footnotes

1 Given laws based upon the United Nations Paris Climate Agreement (COP21, 2016).

2 Health care is not the only sector in which environmental sustainability is intrinsically connected to their basic values. It obviously applies also to organizations or sectors that have planetary health or the protection of the natural environment as their ultimate aim anyway. But there are more institutions (analogous to solidaristic health care systems) for which sustainability is a constitutive element of their ultimate values. Think of the FAO (Food and Agriculture Organization of the United Nations) given its commitment to food security for all. For most public or private organizations, it is less clear that sustainability would be an *intrinsic* – hence inevitable value. This of course does not preclude the possibility that many such organizations can and do decide to embrace sustainability as a key value, or that they internalize the norms for sustainable production that are imposed upon them.

3 Cristina Richie offers a provocative argument for a green bioethics, which implies that health systems should be much more restricted to basic health care [9].

4 Ideals are in this way like imperfect duties. If I decide not to help a specific person in need, this does not necessarily imply a violation of the duty of beneficence – imperfect duties allow for personal choices about when, where and in what ways to act in line with duty. The imperfect or wide obligation of beneficence however does rule out being indifferent to other people’s needs.
